# Seroconversion to *Brucella* spp. and *Toxoplasma gondii* in Sheep and Goats in Dohuk Province, Iraq and Its Association with Pregnancy Loss

**DOI:** 10.3390/ani11030836

**Published:** 2021-03-16

**Authors:** Ali Al Hamada, Ihab Habib, Mieghan Bruce, Anne Barnes, Ian D. Robertson

**Affiliations:** 1 School of Veterinary Medicine, College of Science, Health, Engineering and Education, Murdoch University, Perth 6150, Australia; Gaddan76@gmail.com (A.A.H.); Mieghan.Bruce@murdoch.edu.au (M.B.); i.robertson@murdoch.edu.au (I.D.R.); 2 Department of Theriogenology, College of Veterinary Medicine, University of Mosul, Mosul 41002, Iraq; 3 Department of Veterinary Medicine, College of Food and Agriculture, United Arab Emirates University (UAEU), P.O. Box 15551, Al Ain, Abu Dhabi, United Arab Emirates; 4 Centre for Animal Production and Health, Food Futures Institute, Murdoch University, Perth 6150, Australia; 5 China-Australia Joint Research and Training Center for Veterinary Epidemiology, Huazhong Agricultural University, Wuhan 430070, China

**Keywords:** brucellosis, toxoplasmosis, small ruminants, abortion, seroprevalence

## Abstract

**Simple Summary:**

Brucellosis and toxoplasmosis cause economic losses in small ruminants, notably through abortions. Both *Brucella melitensis* and *Toxoplasma gondii* are important zoonotic agents with infection of the former arising from contact with infected small ruminants or their products and the latter through ingestion of tissue cysts in undercooked/raw meat products of livestock or from oocysts from cats. In Iraq, factors which influence reproductive failure in small ruminants are mostly unknown; however, of the many endemic diseases present, brucellosis and toxoplasmosis are considered important in reducing reproductive output and productivity. This study is part of an integrated research project aiming at understanding the epidemiology of reproductive diseases in small ruminants in northern Iraq. We present in this work a prospective cohort study aiming to determine the incidence risk of seroconversion to *Brucella* and *Toxoplasma* and the association of seroconversion with the reproductive outcome in pregnant sheep and goats in Dohuk, northern Iraq.

**Abstract:**

In this study, sera from 240 small ruminants (192 sheep and 48 goats) belonging to 12 farms in Dohuk Province, northern Iraq, were collected on two occasions to investigate the incidence risk of seroconversion to *Brucella* spp. and *Toxoplasma gondii*. All selected animals were confirmed pregnant (approximately 2 months pregnant) by ultrasound examination at the time of the first blood collection. A second ultrasound examination and blood sampling were undertaken two months after the initial scanning/sampling. Antibodies to *Brucella* were tested using the Rose Bengal Test (RBT) and an indirect enzyme-linked immunosorbent assay (iELISA), and the results were interpreted in series. The Latex Agglutination Test (LAT) and an indirect enzyme-linked immunosorbent assay (iELISA) were also used in series to confirm the presence of antibodies to *T. gondii*. The seroprevalence for *Brucella* and *Toxoplasma* increased significantly between the two sampling times (*p* = 0.0003 and 0.03 in first and second sampling, respectively). The incidence risk of seroconversion to *Brucella* over the two months was 10.6% (95% CI: 6.9–15.3) and 7.3% (95% CI: 4.3–11.6) for *Toxoplasma*. Animals that seroconverted to *Brucella* were 2.9 times more likely to lose their pregnancy (95% CI: 1.6–5.5) than animals that remained seronegative; however, seroconversion to *Toxoplasma* had no significant impact on loss of pregnancy. This study is the first reported investigation on the association of seroconversion to *Brucella* and *Toxoplasma* with the reproductive outcome of pregnant sheep and goats in northern Iraq. Brucellosis and toxoplasmosis continue to negatively impact small ruminants’ reproductive performance and compromising food security in Iraq. It is hoped that this study will assist the development of a better-informed economic model to estimate *Brucella* and *Toxoplasma* burden in small animals in northern Iraq, and such a model could be used to validate the impact of various potential intervention programs in.

## 1. Introduction

In Iraq, there are an estimated 6.6 million sheep and 1.3 million goats, representing a valuable source of meat, milk, and fibre production, and providing income and job security to people working across the agricultural sector [1]; however, disease is a significant challenge facing the small ruminant sector in the country [2]. Like many endemic animal diseases, brucellosis and toxoplasmosis are poorly managed and controlled in Iraq as a consequence of poor veterinary infrastructure [3,4]. Infection with *Toxoplasma gondii* may result in early embryonic death and resorption or foetal mummification [5,6]. The infection outcome is influenced by the stage of pregnancy at which the ewe/doe becomes infected; the earlier infection occurs during the gestation period, the more severe the consequence [5]. As well as the loss of offspring, there is also reduced milk production, resulting in a major economic loss to farmers and the general community [7]. In small ruminants, brucellosis from infection with *Brucella melitensis* also results in economic losses through abortions, decreased milk production and infertility [8]. Sheep and goats are considered the primary hosts for *B. melitensis*; however, affected females usually show no clinical signs until late gestation [9]. Both *B. melitensis* and *T. gondii* are also important zoonotic agents with infection of the former arising from contact with infected small ruminants or their products [10], and the latter through ingestion of tissue cysts in undercooked/raw meat products of livestock or from oocysts of cats [11]. 

In Iraq, factors which are associated with reproductive failure in small ruminants are mostly unknown; however, of the many endemic diseases present, brucellosis and toxoplasmosis are considered important in reducing reproductive output and productivity [12,13]. Although recent studies undertaken in Iraq have highlighted a significant association between seropositivity to *Brucella* or *Toxoplasma* and abortion in sheep and goats [2,14], these were based on cross-sectional studies with their accompanying potential biases, in particular identifying whether infection as measured by a seropositive reaction occurred prior to or after pregnancy loss. This study is part of an integrated research project aiming at understanding the epidemiology of reproductive diseases in small ruminants in northern Iraq. We explored seropositivity patterns and risk factors of *Brucella* and *Toxoplasma* in sheep and goats in Dohuk Province in northern Iraq in previous work [2,14]. Moving forward, we present in this work a prospective cohort study aiming to determine the incidence risk of seroconversion to *Brucella* and *Toxoplasma* and the association of seroconversion with the reproductive outcome in pregnant sheep and goats in Dohuk. We anticipate that this study’s findings will provide objective evidence that could be used for the future development of a well-informed economic modelling evaluation of *Brucella* and *Toxoplasma* burden in small animals in Iraq, and such a model could be used to validate the impact of various potential intervention programs.

## 2. Materials and Methods

### 2.1. Study Area and Selection Criteria

Dohuk Province contains approximately 1,000,000 (one million) small ruminants (unpublished records from Dohuk Veterinary Hospital). Dohuk is the most northern province in Iraq and is located in a very sensitive area, bordering conflict and war zones in Syria and Turkey. Conducting and collecting the study samples and epidemiological data from local sheep and goats was challenging, given security concerns and limited infrastructure in the study setting [15]. Six of the seven districts in the province were included in this study (one district could not be accessed due to security concerns). All 12 sub-districts of the six districts were included in this study. There was no complete sampling frame (structured list of farms and contacts) that could be supplied from local veterinary authorities. informal sampling frame of farms with small ruminants was adopted in this study, where local community leaders in each district were asked to provide the names of farmers who owned sheep and/or goats in each sub-district. From each sub-district, one farm was selected for inclusion in the study; these farms’ owners were approached for inclusion in the study, based on whether the householder was present and willing to participate in the study. In total, 12 (one from each of the 12 sub-districts) farms representing mixed flocks of Awassi sheep and local Iraqi goats were included in this study. Animals were pregnancy tested by ultrasonographic examination (as will be explained in the following section), and 20 animals that were approximately 2 months pregnant were then randomly selected from each participating flock for inclusion in the study (until a total of 240 animals were obtained; 192 sheep, 48 goats).

### 2.2. Ultrasonography Examinations

All animals were scanned transabdominally using an ultrasound scanner equipped with a 3.5 MHz Linear array transducer (Aloka SSD-500, Aloka Co.Ltd., and Tokyo, Japan). All the selected animals were scanned at both blood collection time points (approximately 2 and 4 months of gestation). The animals were scanned in dorsal recumbency without shaving the ventral abdominal wall. The transducer was applied to both sides of the inguinal region’s hairless area after the application of the coupling gel. An animal was confirmed pregnant by identifying a foetal heartbeat or visible movement of a foetus(es) during the scanning. The time spent on each animal to reach a diagnosis of pregnancy status was between 5 and 10 min.

### 2.3. Serological Analyses and Immunization Context 

In the study setting in northern Iraq, immunization policy against *Brucella* is based on using the Rev.1 vaccine; a stable live *B. melitensis* attenuated strain administered by the conjunctival route only (not subcutaneous). The immunization program with REV.1 vaccine in Dohuk is dedicated only to young aged small ruminants (for only lambs and kids aged between 3 to 6 months of age). The animals’ ages ranged between 1 and 9 years (median age = 4 years) and were recorded and categorized as ≤4 and >4 years. Animals were maintained within the flocks and managed as per standard practices by the owners/herders.

The selected pregnant animals were blood sampled on two occasions 60 days apart (between 1 May 2017, and 29 August 2017). All animals had previously been vaccinated with Rev. 1 against brucellosis, and the time between the first blood sample collection and the vaccination was confirmed (based on farm records and herders’ feedback) to be more than 6 months; thus, to assure that vaccination would not interfere with the seroconversion status. Approximately 5 mL of blood was collected from each animal by jugular venepuncture and transported to the laboratory on ice within 12 h of collection. Serum was extracted by centrifugation at 3000 rpm for 10 min and stored at −20 °C until testing. Each serum sample was tested for *Brucella* antibodies using a Rose Bengal Test (RBT; VIRCELL, Granada, Spain), and an iELISA (NovaTec, Dietzenbach, Germany). Sera were also tested with a Latex Agglutination Test (LAT; Plasmatic, UK) and iELISA (NovaTec, Dietzenbach, Germany) for antibodies to *Toxoplasma*. The tests were performed according to the manufacturer’s instructions in the laboratory of the Veterinary Hospital of Dohuk Province. An animal was classified as seropositive if both of the relevant tests were positive (RBT and iELISA for *Brucella*, and LAT and iELISA for *Toxoplasma*) (i.e., tests were interpreted in series).

### 2.4. Ethical Approval

This research was approved by the Animal and Human Ethics Committees of Murdoch University (R 2805/15, 2016/002). All procedures were explained to the farmers, and informed verbal consent was obtained from all participants before sampling.

### 2.5. Statistical Analyses

Data were analysed using STATA, Version 15 (Stata Corp LP, College Station, Texas, USA). The seroprevalence and 95% confidence intervals (95% CI) for *Brucella* and *Toxoplasma* were calculated at each sample point. The incidence risk (IR) and 95% confidence intervals (95% CI) were also calculated based on the proportion of animals seroconverting (i.e., animals that yielded a negative test on the initial blood sampling and a positive test on the subsequent test). The association of seroconversion with pregnancy status at the second sampling was assessed by calculating relative risk and their 95% CI. Animal testing was done separately for the first and second samplings, hence the association of species and age group with the seroprevalence and seroconversion (incidence risk) were assessed using Chi square tests for independence or Fisher’s exact tests.

## 3. Results

As shown in Table 1, there were no animals seropositive at the first sampling that were seronegative at the second sampling. At the first sampling time-point, 13 animals (5.4%; 95% CI: 2.9–9.1) were classified as seropositive to *Brucella* and 22 animals (9.2%; 95% CI: 5.8–13.5) seropositive to *T. gondii*. Three animals were seropositive to both *Brucella* and *Toxoplasma* (1.3%; 95% CI: 0.3–3.6) at this sampling. At the second sampling time-point, 37 animals (15.4%; 95% CI: 11.1–20.6) were seropositive to *Brucella* and 38 animals (15.8%; 95% CI: 11.5–21.1) seropositive to *T. gondii*. At this sampling point, five animals were seropositive to both *Brucella* and *Toxoplasma* (2.1%; 95% CI: 0.7–4.8). The seroprevalence to *Brucella* and *Toxoplasma* increased significantly between the two sampling points (*p* = 0.0003 and *p* = 0.027, respectively) (Table 1).

Twenty-four of the 227 animals that were seronegative to *Brucella* at the first sampling seroconverted (incidence risk—10.6% per two months; 95% CI: 6.9–15.3). On the other hand, 16 of 218 animals were seronegative to *T. gondii* at the first sampling seroconverted (IR—7.3% per two months; 95% CI: 4.3–11.6). There was no significant difference between age groups (≤4 and >4 years) for seroconversion to *Brucella* (12.4% and 7.3%, respectively; *p* = 0.23). However, more older animals (>4 years) seroconverted to *Toxoplasma* (14.8%) than younger animals (≤4 years) (2.9%) (*p* = 0.002). There was no significant difference in the IR over the two-month period between sheep and goats for seroconversion to *Brucella* (sheep—10.3%, 95% CI: 6.3 – 15.6; and goats—11.9%, 95% CI: 4.0–25.6) and *Toxoplasma* (sheep—8.1%, 95% CI: 4.5 – 13.2; and goats—4.4%, 95% CI: 0.5–15.1) (*p* = 0.76, 0.53, respectively), consequently data for both species were combined.

During the study, 39 animals (16.3%, 95% CI: 11.8–21.5) (32 sheep, 7 goats) lost their pregnancy. For *Brucella* this comprised 26 (22 sheep, 4 goats) animals that were seronegative at both sampling points (12.8%, 95% CI: 8.5–18.2), 9 (7 sheep, 2 goats) animals that seroconverted (37.5%, 95% 18.8–59.4) and four animals that were seropositive at both samplings (30.8%, 95% CI: 9.1–61.4) (three sheep, one goat). Small ruminants that seroconverted to *Brucella* were 2.9 times (Relative Risk (RR) = 2.9, 95% CI: 1.6–5.5) more likely to lose their pregnancy than animals that remained seronegative (Table 1).

Seroconversion to *Toxoplasma* was not significantly associated with the number of small ruminants that lost their pregnancy (Table 1). Only 15 of the 39 animals that lost their pregnancy (38.5%, 95% CI: 23.4–55.4) had at least one seropositive result to *Toxoplasma* or *Brucella* (including one sheep seroconverted to both pathogens).

## 4. Discussion

In this work, we adopted a prospective cohort study methodology to generate locally informative incidence risk parameters that could be fed into a subsequent model-based approach that informs evidence-based management of *Brucella* and *Toxoplasma* in northern Iraq. The study setting was very challenging due to security and ethnic conflicts running around the fieldwork area [15]. The study had experienced some limitations; for instance, ultrasonography and blood collection were maintained at approximately 2 and 4 months of gestation, and it was not possible to extend the fieldwork period (due to security concerns) to identify further if the foetal losses of females could have occurred after the second blood collection. Poor record-keeping, or absence of farm records, was evident in many of the visited farms in Dohuk. Therefore, it was not practically possible to capture the exact time of pregnancy and the exact date of vaccination for some animals. As an alternative solution, we had to rely on the owners/herders’ word of mouth, which could have introduced some recall bias. It is not uncommon to rely on expert locals’ opinions while researching in resources limited countries, where data and records might not be available nor accessible, as is the case in industrialized countries [16]. There is also a limitation in this work associated with the nature of the iELISA technique, since the classification of positive versus negative results is based on cut-off value, and also small fluctuation in the reactivity of the serum could lead to changes in the outcome which sometimes might have no real biological meaning. Thus, in future work, it is recommended to include DNA-based detection methods in parallel with serology-based screening to reduce testing misclassification. In this study, lack of local resources and access to logistic support in the field limited our capability to confirm some of the findings using the DNA detection of *Brucella* or *Toxoplasma* by PCR-based methods.

Despite limitations, this is the first study conducted in Iraq investigating the incidence of *Brucella* and *Toxoplasma* infection and the association between seroconversion to these pathogens and maintenance of pregnancy in small ruminants. Previous studies were undertaken in Iraq, and most international studies on brucellosis and toxoplasmosis have focused on cross-sectional studies [17,18,19]. Despite their well-established epidemiological value, cross-sectional studies have potential biases, including biased sampling, an inability to confirm the occurrence of a disease outcome with the timing of infection or seroconversion, i.e., proving causation, and self-reporting by herders/owners of disease outcomes [20,21]. Although cohort studies are more expensive and time-consuming, they overcome these biases [22,23]. The overall incidence of abortion in the current study (16.3%) was comparable to that (20%) reported by Al-Talafhah et al. [24] in Awassi sheep in Jordan. However, the pregnancy losses in the group that seroconverted to brucellosis in the current study (37.5%) were significantly higher than that (13%) in the study of Al-Talafhah et al. [24], although in the latter study infection was confirmed by culture.

In this study, seroconversion to brucellosis was associated with a higher incidence of pregnancy loss than animals that remained seronegative on both tests (RR = 2.9; 95% CI: 1.6–5.5). This is not surprising as one of the most commonly reported clinical signs of infection with *Brucella* in small ruminants is abortion/loss of pregnancy. Small ruminants generally abort due to infection with *Brucella* in the second half of their gestational period, with most aborting during the last third, after day 98 of gestation [25,26,27]. Animals infected with *Brucella* are often seronegative for an extended period until the infection is activated by stress or other factors, including pregnancy [28]. It has been hypothesized that pregnant animals have an increased susceptibility to infection with *Brucella* due to physiological and immunological changes associated with the pregnancy [29]. The presence of erythritol in the placenta of small ruminants is responsible for the localization of *B. melitensis* to this site with subsequent accumulation of large numbers of bacteria, eventually leading to abortion of the foetus [30]. 

Previous research has reported negative serology with the RBT four to six months after conjunctival vaccination with Rev 1 [31], which is the route of vaccination adopted in small ruminants in Dohuk, and across Iraq in general, where the immunisation programme is directed towards only lambs and kids from 3 to 6 months of age and the route of vaccination is through the conjunctival [32]. Vaccination of young aged small ruminants via conjunctival routes is traditionally adopted to overcome several problems experienced with subcutaneous vaccination in older animals [10,33]. One of the critical problems in the vaccination of adult aged small ruminants is the level of antibody responses which are induced by the vaccine, and these may stay for a long time and cause seropositivity of vaccinated animals in routine serological tests, and this causes interfering with detection of the infected ones [10,34,35]. This makes the simultaneous implementation of vaccination and test and slaughter impossible since vaccinated animals are falsely diagnosed as infected [34]. Moreover, the vaccine may induce abortion, and also, the vaccine strain excretion through the milk and vaginal discharges may happen [10,36]. When Rev.1 vaccine in sheep and goats is administered through the conjunctival route, the protection conferred is the same as that induced by the subcutaneous method, but the serological response evoked is significantly reduced [37,38]. In the present study, we tried our best to limit any bias that could be introduced by *Brucella* vaccination effect on seroconversion results. The key inclusion criterion for female animals to be included in the study sample was that the time between vaccination with REV.1 and the first blood sample was more than 6 months. 

In the current study, as antibodies to *Brucella* were confirmed by testing with an RBT and an iELISA and the results interpreted in series, it is assumed that seropositivity resulted from natural infection rather than a serological response to prior vaccination. Aborted foetal and placental material resulting from infection with *Brucella* can lead to significant contamination of the environment resulting in disease spread to other ruminants [39]. This large environmental burden may overcome the immunity induced by vaccination [40]; however, in the current study location, it is also possible that the vaccine was not administered appropriately or was not maintained in a manner to confirm its efficacy due to poor veterinary services and infrastructure in Iraq [41]. Furthermore, the unregulated movement of small ruminants within Iraq and neighbouring countries and the lack of a nationwide mass vaccination campaign against brucellosis in Iraq are likely to result in the mixing of infected, naïve, and vaccinated animals within a flock. The practice of co-grazing of sheep and goats from different flocks in Dohuk Province further increases the likelihood of cross-infection between flocks [2]. Unfortunately, in this study, it was impossible to culture the *Brucella* species associated with seropositivity in the small ruminants. Although it is possible that other *Brucella* species, such as *B. abortus*, may have been responsible for the observed seroconversion, other studies conducted in the region have reported that *B. melitensis* is the most important species affecting small ruminants [24].

In contrast to brucellosis, although the prevalence of *Toxoplasma* increased during the study, there was no significant association between seroconversion and loss of pregnancy with only one animal losing its pregnancy and seroconverting to this protozoan. This may mean that the number of infected cats on the source farms is meagre. *T. gondii* infection is not commonly spread among the small ruminants in Dohuk province, which most likely shows low transmission chance to humans through small ruminant consumption as a food of animal origin [42]. Notably, some researchers have reported that up to 20 million oocysts can be shed by infected cats [43], and an infective dose of only 200 oocysts is required to induce abortion in sheep [44,45]. Following infection with *T. gondii*, small ruminants develop humoral and cell-mediated immune responses against the parasite that provides adequate protection against clinical disease in subsequent pregnancies [5]. In the current study the proportion of *Toxoplasma* seronegative animals that lost their pregnancy (17.8%) was higher, although not significantly, than for those that seroconverted or had two positive test results. This would indicate that infection with *T. gondii* is not a significant cause of pregnancy loss in the flocks from which the animals were sourced and most likely in all flocks of Dohuk Province. 

Only 15 of the 39 animals that lost their pregnancy (38.5%) had at least one positive result for *Toxoplasma* or *Brucella*, indicating that other causes, either non-infectious or infectious, were associated with these losses. Pregnancy loss is often a result of multifactorial aetiologies [46], and other causes of loss in sheep and goats, such as infection with *Chlamydophila abortus*, Border Disease Virus, *Neospora caninum*, or *Coxiella burnetti* [47,48,49,50], or non-infectious causes, such as deficiencies in vitamin A, E, selenium, zinc, copper, phosphorus or magnesium, may have been responsible for these losses [46].

## 5. Conclusions

To our knowledge, this was the first prospective study to analyse the relationship between seroconversion to *Brucella* and *Toxoplasma* and loss of pregnancy in small ruminants in Iraq. Despite some limitations, the results of this work are valuable to calculate and/or model the financial impact of these two pathogens on the productivity of small ruminants and to investigate the economic value of implementing control measures, including mass vaccination programs, in the region.

## Figures and Tables

**Table 1:** Serological response to *Brucella* and *Toxoplasma* in 240 pregnant sheep and goats at two sampling points and the effect on pregnancy status.

Initial Sample	Second Sample	Number of Animals (%; 95% CI)	Number of Animals Lost Their Pregnancy (%; 95% CI)	Relative Risk (RR) for Pregnancy Loss (%; 95% CI)
*Brucella*				
Seronegative	Seronegative	203 (84.6; 79.4–88.9)	26 (12.8; 8.5–18.2)	1.0 (Reference category)
Seronegative	Seropositive	24 (10.0; 6.5–14.5)	9 (37.5; 18.8–59.4)	2.9 (1.6, 5.5)
Seropositive	Seropositive	13 (5.4; 2.9–9.1)	4 (30.8 9.1–61.4)	2.4 (1.0, 5.9)
*Toxoplasma*				
Seronegative	Seronegative	202 (84.2; 78.9–88.5)	36 (17.8; 12.8–23.8)	1.0 (Reference category)
Seronegative	Seropositive	16 (6.7; 3.9–10.6)	1 (6.3; 0.2–30.2)	0.4 (0.1, 2.4)
Seropositive	Seropositive	22 (9.2; 5.8–13.5)	2 (9.1 (1.1–29.2)	0.5 (0.1, 2.0)

## Data Availability

The data used to support the findings of this study are included within the article.
